# 2,3,5,6-Tetra­methoxy­piperazine-1,4-dicarbaldehyde

**DOI:** 10.1107/S1600536809035259

**Published:** 2009-09-05

**Authors:** Sayed Mojtaba Moosavi, Amir Taheri

**Affiliations:** a Department of Chemistry, Imam Hossein University, Tehran, Iran

## Abstract

The asymmetric unit of the title compound, C_10_H_18_N_2_O_6_, contains two halves of two independent centrosymmetric mol­ecules with almost identical conformations. Weak inter­molecular C—H⋯O hydrogen bonds consolidate the crystal packing.

## Related literature

For details of the synthesis, see: Ferguson (1968*a* ▶,*b* ▶). For a closely related compound with acetyl substituents, see: Vedachalam *et al.* (1991 ▶). For anomeric inter­actions, see: Reed & Schleyer (1988 ▶). For glycoside structures, see: Schleifer *et al.* (1990 ▶).
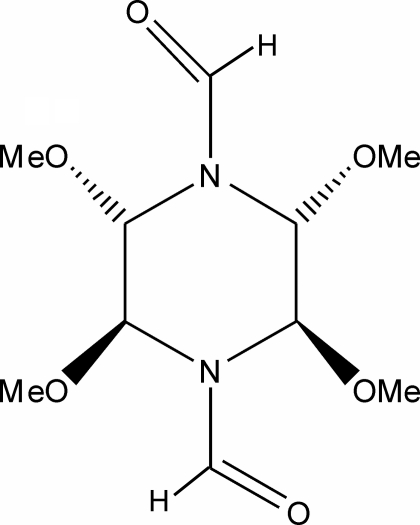


## Experimental

### 

#### Crystal data

C_10_H_18_N_2_O_6_
*M* *_r_* = 262.26
Monoclinic, 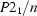

*a* = 14.331 (4) Å
*b* = 6.6044 (18) Å
*c* = 14.332 (4) Å
β = 114.800 (3)°
*V* = 1231.4 (6) Å^3^
*Z* = 4
Mo *K*α radiation
μ = 0.12 mm^−1^
*T* = 120 K
0.5 × 0.05 × 0.05 mm

#### Data collection

Bruker SMART 1000 CCD area-detector diffractometer
Absorption correction: multi-scan (*SADABS*; Sheldrick, 1998 ▶) *T*_min_ = 0.980, *T*_max_ = 0.995
12344 measured reflections
2958 independent reflections
2242 reflections with *I* > 2σ(*I*)
*R*_int_ = 0.037

#### Refinement

*R*[*F* ^2^ > 2σ(*F* ^2^)] = 0.059
*wR*(*F* ^2^) = 0.127
*S* = 1.00
2958 reflections
168 parameters
H-atom parameters constrained
Δρ_max_ = 0.56 e Å^−3^
Δρ_min_ = −0.30 e Å^−3^

### 

Data collection: *SMART* (Bruker, 1998 ▶); cell refinement: *SAINT-Plus* (Bruker, 1998 ▶); data reduction: *SAINT-Plus*; program(s) used to solve structure: *SHELXTL* (Sheldrick, 2008 ▶); program(s) used to refine structure: *SHELXTL*; molecular graphics: *SHELXTL*; software used to prepare material for publication: *SHELXTL*.

## Supplementary Material

Crystal structure: contains datablocks I, global. DOI: 10.1107/S1600536809035259/cv2606sup1.cif

Structure factors: contains datablocks I. DOI: 10.1107/S1600536809035259/cv2606Isup2.hkl

Additional supplementary materials:  crystallographic information; 3D view; checkCIF report

## Figures and Tables

**Table 1:** Hydrogen-bond geometry (Å, °)

*D*—H⋯*A*	*D*—H	H⋯*A*	*D*⋯*A*	*D*—H⋯*A*
C5—H5*C*⋯O6	0.96	2.53	3.260 (4)	133
C3—H3*A*⋯O1^i^	0.93	2.46	3.371 (3)	167
C8—H8*A*⋯O4^ii^	0.93	2.47	3.364 (4)	163
C10—H10*B*⋯O3^iii^	0.96	2.60	3.208 (3)	122

Symmetry codes: (i) 
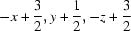
; (ii) 
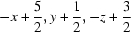
; (iii) 
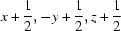
.

**Fig. 1.**
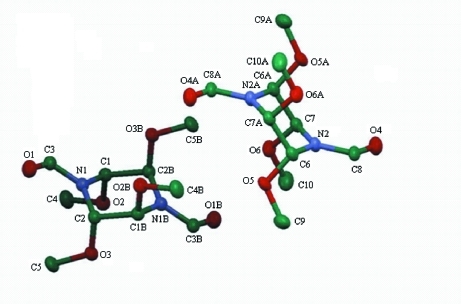
Two independent molecules of (I) showing the atomic numbering and 50% probability displacement ellipsoids [symmetry codes: (A) 1 - x, 1 - y , 1 - z; (B) 2 - x , 1 - y , 1 - z]. H atoms omitted for clarity.

**Fig. 2.**
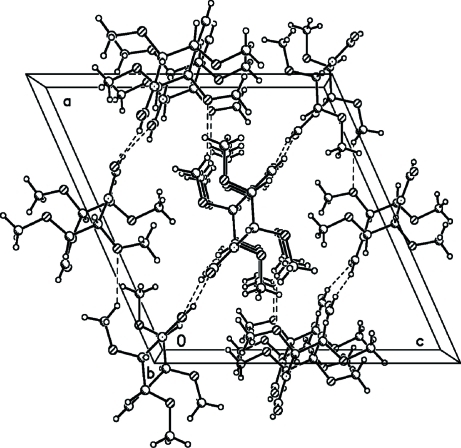
A portion of the crystal packing viewed along crystallographic axis b. Dashed lines denote hydrogen bonds.

## supplementary crystallographic information

### Comment

The molecule of 1,4-diformyl-2,3,5,6-tetra-methoxypiperazine (Ferguson,1968*a*, b) (I), contains two formamyl groups in the two contrary-sides of the six-membered ring and four methoxy groups in the same axial situations. There are four asymmetric carbon atoms, S, S and *R*, *R* configuration which are mirror images of each other and diastereoisomer. The skeleton is symmetric by a central symmetry. The crystal contains two halves of two independent centrosymmetric molecules with almost identical conformations, which are comparable with similar molecule with acetyl substitutions (Vedachalam *et al.*, 1991).

Despite the presence of six O and two N atoms carrying lone-pair electrons potentially are available for hydrogen-bond formation, there are, in fact, no powerful intramolecular C—H···N or C—H···O hydrogen bonds, rather some week C—H···O intermolecular interactions are observed (Table 1, Fig. 2).

The Properties of the anomeric effect in the infected system, *n*_O_→σ*_C—N _interactions are recognizable (Reed & Schleyer, 1988), The O—C bond is shorter than the C—N bond and the stability of *gauche* (axial) forms is definitely more than anti (equatorial) ones (Figure 1). This electron transfer results in lengthening the exocyclic anomeric C—O bonds, in the endocyclic contraction of the C—N bonds by increasing its double-bond character in the opening of the O—C—N angle as compared with its normal tetrahedral value. It can be considered that aa, ag and gg conformations of the C—O—C—N—C moieties, where a = anti (antiperiplanar) and g = *gauche* (synclinal) which aa and ag refer to equatorial and gg to axial. All of the Methoxy groups are in the gg conformations, comparable with gg in glycoside conformations that are more stable than aa, equatorial conformations (Schleifer *et al.*, 1990). The acetyl groups as substitutions select axial positions than equatorials (Vedachalam *et al.*, 1991).

In (I) *via* resonation of N-lone-pairs with *pi*-electrons of the carbonyl double-bond, N-pyramidalities (about 360°) are larger than natural forms (about 320°) at the same of molecule with acetyl substitutions (Vedachalam *et al.*, 1991).

### Experimental

1,4-Diformyltetrachloropiperazine (7.04 g,0.04 mol) (Ferguson, 1968*a*,b) were mixed with barium carbonate (78 g) and absolute methanol (32 ml, 0.79 mol) were added to the mixture and heated at 313 K for 3 h with stirring. The barium carbonate was filtered off and the methanol solution evaporated. The solid residue was extracted by 20 ml of hot chloroform and the extract evaporated to give white precipitation and washed with a little cold water and dried in oven by 303 K of temperature affording 1.83 g (70% yield) of (I). This solid was crystallized by hot mixture of methanol and benzene (m.p. 469 K). ^1^HNMR (CDCl_3_): δ_H_ 8.47 (s, 2H, CH), 5.29 (d, 2H, CH), 4.82 (d, 2H, CH), 3.21 (m, 12H,CH). ^13^C NMR (CDCl_3_): δ_C_165 (2CH), 83.4 (2CH), 79.1(2CH), 54.5 (6CH), 56.2 (6CH).

### Refinement

All H atoms were geometrically positioned (C—H 0.93 - 0.98 Å) and refined as riding, with *U*_iso_(H) = 1.2-1.5 *U*_eq_(C).

### Crystal data

**Table:**

C_10_H_18_N_2_O_6_	*F*(000) = 560
*M**_r_* = 262.26	*D*_x_ = 1.415 Mg m^−^^3^
Monoclinic, *P*2_1_/*n*	Mo *K*α radiation, λ = 0.71073 Å
Hall symbol: -P 2yn	Cell parameters from 1254 reflections
*a* = 14.331 (4) Å	θ = 2–25°
*b* = 6.6044 (18) Å	µ = 0.12 mm^−^^1^
*c* = 14.332 (4) Å	*T* = 120 K
β = 114.800 (3)°	Needle, colourless
*V* = 1231.4 (6) Å^3^	0.5 × 0.05 × 0.05 mm
*Z* = 4	

### Data collection

**Table:**

Bruker SMART 1000 CCD area-detector diffractometer	2958 independent reflections
Radiation source: fine-focus sealed tube	2242 reflections with *I* > 2σ(*I*)
graphite	*R*_int_ = 0.037
φ and ω scans	θ_max_ = 28.0°, θ_min_ = 1.6°
Absorption correction: multi-scan (*SADABS*; Sheldrick, 1998)	*h* = −18→18
*T*_min_ = 0.980, *T*_max_ = 0.995	*k* = −8→8
12344 measured reflections	*l* = −18→18

### Refinement

**Table:**

Refinement on *F*^2^	Primary atom site location: structure-invariant direct methods
Least-squares matrix: full	Secondary atom site location: difference Fourier map
*R*[*F*^2^ > 2σ(*F*^2^)] = 0.059	Hydrogen site location: inferred from neighbouring sites
*wR*(*F*^2^) = 0.127	H-atom parameters constrained
*S* = 1.00	*w* = 1/[σ^2^(*F*_o_^2^) + (0.0108*P*)^2^ + 2.6266*P*] where *P* = (*F*_o_^2^ + 2*F*_c_^2^)/3
2958 reflections	(Δ/σ)_max_ = 0.001
168 parameters	Δρ_max_ = 0.56 e Å^−^^3^
0 restraints	Δρ_min_ = −0.29 e Å^−^^3^

### Special details

**Table:**

Geometry. All e.s.d.'s (except the e.s.d. in the dihedral angle between two l.s. planes) are estimated using the full covariance matrix. The cell e.s.d.'s are taken into account individually in the estimation of e.s.d.'s in distances, angles and torsion angles; correlations between e.s.d.'s in cell parameters are only used when they are defined by crystal symmetry. An approximate (isotropic) treatment of cell e.s.d.'s is used for estimating e.s.d.'s involving l.s. planes.
Refinement. Refinement of *F*^2^ against ALL reflections. The weighted *R*-factor *wR* and goodness of fit *S* are based on *F*^2^, conventional *R*-factors *R* are based on *F*, with *F* set to zero for negative *F*^2^. The threshold expression of *F*^2^ > σ(*F*^2^) is used only for calculating *R*-factors(gt) *etc*. and is not relevant to the choice of reflections for refinement. *R*-factors based on *F*^2^ are statistically about twice as large as those based on *F*, and *R*- factors based on ALL data will be even larger.

### Fractional atomic coordinates and isotropic or equivalent isotropic displacement parameters (Å^2^)

**Table:**

	*x*	*y*	*z*	*U*_iso_*/*U*_eq_	
O1	0.67186 (15)	0.1494 (3)	0.66884 (16)	0.0354 (5)	
O2	0.62334 (13)	0.7282 (3)	0.49476 (14)	0.0279 (4)	
O3	0.55403 (13)	0.3233 (3)	0.39458 (14)	0.0291 (4)	
O4	1.17420 (15)	0.1502 (3)	0.66699 (16)	0.0354 (5)	
O5	0.99381 (14)	0.7197 (3)	0.62522 (15)	0.0311 (5)	
O6	0.89855 (13)	0.3088 (3)	0.55478 (15)	0.0322 (5)	
N1	0.59356 (15)	0.4210 (3)	0.56729 (16)	0.0237 (5)	
N2	1.06918 (15)	0.4199 (4)	0.59207 (17)	0.0245 (5)	
C1	0.58449 (18)	0.6410 (4)	0.5610 (2)	0.0239 (5)	
H1A	0.6218	0.6977	0.6300	0.029*	
C2	0.52907 (18)	0.2971 (4)	0.47925 (19)	0.0245 (5)	
H2A	0.5365	0.1541	0.4993	0.029*	
C3	0.66071 (19)	0.3326 (4)	0.6564 (2)	0.0260 (6)	
H3A	0.7002	0.4165	0.7108	0.031*	
C4	0.73438 (19)	0.7406 (5)	0.5418 (2)	0.0332 (7)	
H4A	0.7577	0.7963	0.4934	0.050*	
H4B	0.7628	0.6077	0.5618	0.050*	
H4C	0.7563	0.8262	0.6013	0.050*	
C5	0.6535 (2)	0.2490 (5)	0.4132 (2)	0.0360 (7)	
H5A	0.6669	0.2731	0.3539	0.054*	
H5B	0.6565	0.1063	0.4268	0.054*	
H5C	0.7041	0.3176	0.4715	0.054*	
C6	1.06031 (19)	0.6398 (4)	0.5843 (2)	0.0259 (6)	
H6A	1.1287	0.6991	0.6214	0.031*	
C7	0.98182 (18)	0.2938 (4)	0.5274 (2)	0.0268 (6)	
H7A	1.0040	0.1522	0.5327	0.032*	
C8	1.16039 (18)	0.3332 (4)	0.6578 (2)	0.0259 (6)	
H8A	1.2143	0.4181	0.6972	0.031*	
C9	1.0398 (2)	0.7262 (5)	0.7347 (2)	0.0384 (7)	
H9A	0.9897	0.7693	0.7587	0.058*	
H9B	1.0962	0.8199	0.7579	0.058*	
H9C	1.0644	0.5939	0.7614	0.058*	
C10	0.9185 (2)	0.2250 (6)	0.6521 (2)	0.0412 (8)	
H10A	0.8587	0.2393	0.6657	0.062*	
H10B	0.9753	0.2948	0.7041	0.062*	
H10C	0.9351	0.0841	0.6526	0.062*	

### Atomic displacement parameters (Å^2^)

**Table:**

	*U*^11^	*U*^22^	*U*^33^	*U*^12^	*U*^13^	*U*^23^
O1	0.0309 (10)	0.0386 (13)	0.0360 (11)	0.0059 (9)	0.0134 (9)	0.0068 (9)
O2	0.0191 (8)	0.0373 (11)	0.0310 (10)	−0.0045 (8)	0.0141 (7)	0.0020 (8)
O3	0.0198 (8)	0.0421 (12)	0.0274 (9)	0.0021 (8)	0.0119 (7)	−0.0010 (9)
O4	0.0257 (9)	0.0393 (13)	0.0407 (12)	0.0066 (9)	0.0134 (9)	0.0067 (10)
O5	0.0205 (8)	0.0430 (12)	0.0334 (10)	0.0052 (8)	0.0148 (8)	−0.0060 (9)
O6	0.0167 (8)	0.0475 (13)	0.0341 (10)	0.0017 (8)	0.0124 (8)	0.0062 (9)
N1	0.0180 (9)	0.0285 (12)	0.0258 (11)	−0.0011 (8)	0.0103 (8)	0.0003 (9)
N2	0.0152 (9)	0.0295 (12)	0.0284 (11)	0.0010 (8)	0.0088 (8)	−0.0010 (9)
C1	0.0184 (11)	0.0290 (14)	0.0252 (12)	−0.0023 (10)	0.0099 (10)	−0.0007 (11)
C2	0.0177 (11)	0.0320 (15)	0.0256 (12)	−0.0008 (10)	0.0108 (10)	0.0005 (11)
C3	0.0184 (11)	0.0350 (16)	0.0260 (13)	0.0011 (11)	0.0107 (10)	0.0025 (11)
C4	0.0189 (12)	0.0384 (17)	0.0462 (17)	−0.0040 (11)	0.0175 (12)	−0.0005 (14)
C5	0.0220 (13)	0.0527 (19)	0.0379 (16)	0.0055 (13)	0.0172 (12)	−0.0032 (14)
C6	0.0171 (11)	0.0305 (15)	0.0312 (14)	0.0003 (10)	0.0112 (10)	−0.0049 (11)
C7	0.0172 (11)	0.0332 (15)	0.0313 (14)	0.0010 (10)	0.0113 (10)	−0.0033 (11)
C8	0.0153 (11)	0.0367 (16)	0.0275 (13)	0.0023 (10)	0.0107 (10)	0.0042 (12)
C9	0.0395 (16)	0.0463 (19)	0.0335 (15)	0.0073 (14)	0.0193 (13)	−0.0043 (14)
C10	0.0279 (14)	0.056 (2)	0.0452 (18)	0.0047 (14)	0.0203 (13)	0.0170 (16)

### Geometric parameters (Å, °)

**Table:**

O1—C3	1.224 (3)	C2—H2A	0.9800
O2—C1	1.409 (3)	C3—H3A	0.9300
O2—C4	1.447 (3)	C4—H4A	0.9600
O3—C2	1.412 (3)	C4—H4B	0.9600
O3—C5	1.423 (3)	C4—H4C	0.9600
O4—C8	1.223 (3)	C5—H5A	0.9600
O5—C6	1.414 (3)	C5—H5B	0.9600
O5—C9	1.425 (3)	C5—H5C	0.9600
O6—C7	1.408 (3)	C6—C7^ii^	1.519 (4)
O6—C10	1.414 (3)	C6—H6A	0.9800
N1—C3	1.367 (3)	C7—C6^ii^	1.519 (4)
N1—C1	1.458 (4)	C7—H7A	0.9800
N1—C2	1.462 (3)	C8—H8A	0.9300
N2—C8	1.375 (3)	C9—H9A	0.9600
N2—C6	1.458 (4)	C9—H9B	0.9600
N2—C7	1.465 (3)	C9—H9C	0.9600
C1—C2^i^	1.537 (3)	C10—H10A	0.9600
C1—H1A	0.9800	C10—H10B	0.9600
C2—C1^i^	1.537 (3)	C10—H10C	0.9600
			
C1—O2—C4	112.2 (2)	O3—C5—H5B	109.5
C2—O3—C5	113.0 (2)	H5A—C5—H5B	109.5
C6—O5—C9	112.8 (2)	O3—C5—H5C	109.5
C7—O6—C10	113.8 (2)	H5A—C5—H5C	109.5
C3—N1—C1	119.6 (2)	H5B—C5—H5C	109.5
C3—N1—C2	120.6 (2)	O5—C6—N2	113.1 (2)
C1—N1—C2	119.8 (2)	O5—C6—C7^ii^	106.9 (2)
C8—N2—C6	119.6 (2)	N2—C6—C7^ii^	110.5 (2)
C8—N2—C7	120.7 (2)	O5—C6—H6A	108.7
C6—N2—C7	119.6 (2)	N2—C6—H6A	108.7
O2—C1—N1	113.5 (2)	C7^ii^—C6—H6A	108.7
O2—C1—C2^i^	107.1 (2)	O6—C7—N2	112.5 (2)
N1—C1—C2^i^	109.9 (2)	O6—C7—C6^ii^	105.4 (2)
O2—C1—H1A	108.7	N2—C7—C6^ii^	111.1 (2)
N1—C1—H1A	108.7	O6—C7—H7A	109.3
C2^i^—C1—H1A	108.7	N2—C7—H7A	109.3
O3—C2—N1	112.1 (2)	C6^ii^—C7—H7A	109.3
O3—C2—C1^i^	104.8 (2)	O4—C8—N2	123.4 (3)
N1—C2—C1^i^	111.2 (2)	O4—C8—H8A	118.3
O3—C2—H2A	109.5	N2—C8—H8A	118.3
N1—C2—H2A	109.5	O5—C9—H9A	109.5
C1^i^—C2—H2A	109.5	O5—C9—H9B	109.5
O1—C3—N1	123.8 (3)	H9A—C9—H9B	109.5
O1—C3—H3A	118.1	O5—C9—H9C	109.5
N1—C3—H3A	118.1	H9A—C9—H9C	109.5
O2—C4—H4A	109.5	H9B—C9—H9C	109.5
O2—C4—H4B	109.5	O6—C10—H10A	109.5
H4A—C4—H4B	109.5	O6—C10—H10B	109.5
O2—C4—H4C	109.5	H10A—C10—H10B	109.5
H4A—C4—H4C	109.5	O6—C10—H10C	109.5
H4B—C4—H4C	109.5	H10A—C10—H10C	109.5
O3—C5—H5A	109.5	H10B—C10—H10C	109.5
			
C4—O2—C1—N1	−78.1 (3)	C9—O5—C6—N2	77.0 (3)
C4—O2—C1—C2^i^	160.4 (2)	C9—O5—C6—C7^ii^	−161.1 (2)
C3—N1—C1—O2	110.9 (3)	C8—N2—C6—O5	−111.3 (2)
C2—N1—C1—O2	−70.5 (3)	C7—N2—C6—O5	70.6 (3)
C3—N1—C1—C2^i^	−129.1 (2)	C8—N2—C6—C7^ii^	128.9 (2)
C2—N1—C1—C2^i^	49.4 (3)	C7—N2—C6—C7^ii^	−49.1 (3)
C5—O3—C2—N1	66.7 (3)	C10—O6—C7—N2	−67.6 (3)
C5—O3—C2—C1^i^	−172.6 (2)	C10—O6—C7—C6^ii^	171.2 (3)
C3—N1—C2—O3	−114.5 (3)	C8—N2—C7—O6	113.5 (3)
C1—N1—C2—O3	67.0 (3)	C6—N2—C7—O6	−68.5 (3)
C3—N1—C2—C1^i^	128.5 (2)	C8—N2—C7—C6^ii^	−128.7 (2)
C1—N1—C2—C1^i^	−50.0 (3)	C6—N2—C7—C6^ii^	49.4 (3)
C1—N1—C3—O1	178.7 (2)	C6—N2—C8—O4	−178.6 (2)
C2—N1—C3—O1	0.2 (4)	C7—N2—C8—O4	−0.5 (4)

Symmetry codes: (i) −*x*+1, −*y*+1, −*z*+1; (ii) −*x*+2, −*y*+1, −*z*+1.

### Hydrogen-bond geometry (Å, °)

**Table:**

*D*—H···*A*	*D*—H	H···*A*	*D*···*A*	*D*—H···*A*
C5—H5C···O6	0.96	2.53	3.260 (4)	133
C3—H3A···O1^iii^	0.93	2.46	3.371 (3)	167
C8—H8A···O4^iv^	0.93	2.47	3.364 (4)	163
C10—H10B···O3^v^	0.96	2.60	3.208 (3)	122

Symmetry codes: (iii) −*x*+3/2, *y*+1/2, −*z*+3/2; (iv) −*x*+5/2, *y*+1/2, −*z*+3/2; (v) *x*+1/2, −*y*+1/2, *z*+1/2.
